# Laser processing of Ti6Al4V alloy: wetting state of surface and environmental dust effects

**DOI:** 10.1016/j.heliyon.2019.e01211

**Published:** 2019-02-05

**Authors:** B.S. Yilbas, H. Ali, A. Al-Sharafi, H. Al-Qahtani

**Affiliations:** Department of Mechanical Engineering, King Fahd University of Petroleum & Minerals, Dhahran, 31261, Saudi Arabia

**Keywords:** Mechanical engineering

## Abstract

Laser processing of Ti6Al4V alloy surface, via repetitive pulses, is realized incorporating the nitrogen assisting gas. The texture characteristics of the surface and wetting state are analyzed. The free energy of the laser treated surface is estimated. The influence of the dust particles on the treated and untreated surfaces is examined. The solution formed due to water condensate on the dust particles is evaluated. The adhesion of the mud dried solution on the treated and untreated surfaces is assessed through determining the tangential force required for the removal of the solution from the surface. The findings demonstrate that the high power laser repetitive pulse heating results in formation of the hieratically distributed micro/nano pillars on the workpiece surface. The wetting state of the processed surface remains hydrophilic because of the large gap size between the micro/nano pillars. The free energy of the laser textured surface is similar to that obtained for the TiN coated surfaces, which is because of the nitride compounds developed during the processing. The dried liquid solution strongly adheres at the surface and the force needed for removing the dried liquid solution is almost four times of the friction force at the surface. The liquid solution gives rise to locally scattered shallow pit sites on as received surface. This phenomenon does not occur for the laser treated surface, which is related to the passive layer developed on the surface.

## 1 Introduction

Titanium alloys become demanding in many industrial applications due to the requirements of high toughness to mass ratio; however, some of their applications are limited because of poor tribological properties of the alloy surface. Titanium alloys are also used in solar energy harvesting devices, such as in photovoltaic hydrogen production systems [1], in which the surface properties of the alloy remain important. Several methods can be considered towards improving the surface properties and one of the effective methods is to introduce a laser beam treatment of the alloy surface. Laser treatment provides several advantages over the conventional processing methods, which enable the laser treatment process superior to other methods. Laser treatment of metallic surfaces involves with a high temperature processing and the presence of assisting ambient gas influences the resulting surface properties [2]. If oxygen is used in the treated section, high temperature exothermic reactions takes place on the surface and the excessive defects sites are created. However, using assisting gases like nitrogen, argon, or helium minimizes this effect and improves the surface tribological and corrosion properties [3, 4]. Hence, using an assisting gas such as nitrogen not only prevents the surface defects but also forms the nitride species, such as metallic nitride compounds, in the treated region while increasing the surface resistance to wear. Laser treatment alters the surface free energy of the substrate material [5]. However, the surface properties such as free energy and texture characteristics affect the wetting state of the substrate material. This remains critical for the self-cleaning applications or the corrosion resistance of the substrate materials [6, 7]. On the other hand, the climate change causes the dust storms around the Globe, which is more pronounced in the near region of the desert environments. Accumulations of the dust particles have several adverse effects on the surfaces. The dust particles contain alkaline and alkaline earth metal compounds, which can dissolve in the water condensate in humid air ambient. The dissolution of these compounds can cause the chemically active liquid solution spreads on the substrate surface under the gravitational potential energy. In some cases, this causes chemical damages on the substrate surface and upon drying it forms the crystals while strongly adhering on the surface [8]. In addition, the dried liquid solution forms a middle-layer in between the dust particles and the substrate surface while causing enhancement of the dust adhesion on the surfaces. In this case, the removal of the dust particles from the workpiece surface requires excessive efforts. Since the titanium alloys are used in open environments, their surfaces become subject to the dust particle effects in humid air environments. Hence, examination of the laser processing of titanium alloys and effects of the environmental dust particles on the laser treated surface becomes fruitful incorporating the humid air ambient conditions.

Laser processing of Ti-based alloys was investigated by Gupta et al. [9]. They showed that because of non-uniform distribution of the intermetallic compounds, the laser treatment increased the hardness and improved the mechanical properties. A study on the laser shock peening of titanium alloy surface was carried out by Dai et al. [10]. They indicated that the nano-size texturing of the surface was resulted after the shock processing. The influence of laser parameters on surface textures of titanium alloys was considered by Ahuir-Torres et al. [11]. They indicated that, via adjusting the laser frequency and pulse length, the micro-texturing of the surface could be resulted through the ultrafast laser ablation process. The laser heating towards texturing of Ti6Al4V alloy to achieve increasing adhesion in PEEK joints was examined by Henriques et al. [12]. They indicated that laser processing improved the bond strength of the alloy as compared to that of the grit blasting technique. A study on the femtosecond laser nano-patterning of Ti6Al4V alloy was carried out by Rotella et al. [13]. They demonstrated that the periodic texture characteristics could be resulted on the surface via high speed surface ablation. Laser treatment of a titanium alloy and resulting microstructural changes were investigated by Zhou et al. [14]. They showed that laser melting lowered the porosity in titanium alloy. After hot-isostatic pressing, the porosity could be further reduced. The hybrid additive manufacturing of titanium alloy incorporating the laser processing was studied by Yin et al. [15]. They showed that the laser treated titanium alloy exhibited acicular martensitic microstructure. The cold spraying did not result in microstructural changes in the substrate material. However, laser treatment gave rise to increased hardness than that of the cold spraying. The laser treatment of titanium alloy and metallurgical properties of the treated surface were examined by Wang et al. [16]. They demonstrated that titanium carbide did not dissolve in the alloy surface after the laser treatment process. The laser processing of titanium alloy towards improvement of microstructure and hardness was studied by Balla et al. [17]. They showed that α+β phase of the alloy changed to acicular α in β matrix after the laser processing. However, controlling the cooling rates gave rise to grain size arrangements in the alloy. A study on the mechanical behavior of the laser treated Ti6Al4V alloy was carried out by Lu et al. [18]. They demonstrated that the vertical β-grain boundaries caused shear fracture in the workpieces. The shear mode resulted in improvement of the tensile and fracture properties; however, the symmetric necking because of the fine α-grains gave rise to grain boundary deformation. Laser processing of titanium-alloy was examined by Yilbas et al. [19]. The findings showed that TiO, TiO_2_, TiN and TiO_x_N_y_ compounds were developed on the laser processed surface and surface hardness was increased. The surface chemistry of Ti6Al4V workpieces produced via laser processing technique was studied by Vaithilingam et al. [20]. They demonstrated that, in general, the heterogeneity of elemental composition occurred on the treated surface; however, vanadium was observed when the surface was polished mechanically.

The laser surface processing of Ti6Al4V alloy was studied in the early studies [19, 21]; however, the main concerns were to investigate: i) the morphological changes on the resulting surface, and ii) metallurgical variations in the laser treated layer. However, when titanium alloy surface is exposed to the open environment, the dust accumulation on the surface can cause severe damages particularly in humid air ambient. The surface adhesion can be reduced through creating hierarchically distributed texture characteristics on the surface. Hence, in the previous studies [19, 21], the surface wetting state and surface response to the environmental dust adhesion were overlooked and the relevant study was left for future investigation. In addition, the environmental dust particles compose of alkaline and alkaline earth metals, which can dissolve in water condensate and forms chemically active fluid on the laser processed surface. The adhesion of the dried fluid is high and the efforts removing them from the surface becomes large. Hence, laser treatment of Ti6Al4V surface is considered and the dust effects on the surface characteristics, such as morphology and wetting, are examined. The contact angle technique was adopted determining free surface energy of treated Ti6Al4V. The dust collected in outdoor environment is characterized and the bonding of the liquid solution, which is extracted from the dust particles and water condensate mixture through mimicking the outdoor environmental humid air conditions, is assessed.

## 2 Experimental

The CO_2_ laser (LC-ALPHAIII) was utilized treating Ti6Al4V workpiece surfaces. The diameter of laser irradiated spot was 150 μm on the surface. Nitrogen was used as an assisiting gas in the experiments. The laser pulse frequency was 1500 Hz, which produced spots with the overlapping ratio of 70% on the surface. To minimize several repeats of experiments, initial surface treatment tests were realized identifying the proper laser treatment parameters. Hence, the process parameters avoiding surface asperities were incorporated. Hence, the treated surfaces were free from large size asperities after controlling the process parameters. Laser processing conditions are given in Table 1. 3 mm thick Ti6Al4V alloy sheet was selected as the workpieces. The samples were prepared in 10 × 10 × 3 mm^3^: width×length×thickness dimensions. Laser textured surfaces were analyzed via utilizing SEM, EDS, (AFM/SPM), and XRD. Electron microscope was Jeol 6460 and XRD was conducted using Bruker D8 Advanced with CuKα radiation source. The silicon nitride tip was used in atomic force microscope (AFM) analysis. The infrared absorption spectrum was analyzed incorporating Fourier transform infrared spectroscopy was conducted (Bruker – VERTEX70). The micro-hardness tests were realized using microhardness tester (MP-100TC) while incorporating Vickers hardness measurement standard (ASTM C1327-99). The measurements were repeated three times at each location. The assessment of adhesion between the dried mud and the surface was carried out using a linear micro-scratch tester (MCTX-S/N: 01-04300).

**Table 1:** Laser treatment parameters.

Transverse Velocity (cm/s) (mm/min)	Laser Output (W)	Repetition Rate (Hz)	Nozzle Distance (mm)	Focusing Length (mm)	Gass Pressure (kPa)
12	96	1500	1.4	126	575

The residual stress was determined using the XRD data for the polycrystalline structure. The diffraction peak position changed once the workpiece was tilted by an angle ψ and the amount of shift was associated with the residual stress. Hence, the residual stress (σ) could be expressed as [22]:

(1) $$\sigma =\frac{E}{\left(1+\text{υ}\right)Sin^{2}\text{ψ}}\frac{\left(d_{n}-d_{o}\right)}{d_{o}}$$

Here, E represents Young's modulus, υ corresponds to Poisson's ratio, ψ corresponds to the tilting angle, and *d*_*n*_ are the *d* spacing measured at each tilting angle. The calculations were carried out for TiN (111) planes ($2\theta =.36.6^{o}$) while using Eq. (1).

The droplet contact angle was measured via Kyowa (model - DM 501) goniometer adopting the technique used in the early work [23]. The droplet volume had the resolution of 0.1 μl. The dust was gathered from the photovoltaic surfaces in the local area of Dhahran in Saudi Arabia. The collected dust particels were used simulating the effects of the mud, which was formed during the stage of water condensation in the humid air conditions onto the dust particles over the laser processed surface. The experiment was carried out in laboratory condition while resembling the actual liquid solution formed in the outdoor environment. The outdoor tests were carried out determining the mas ratio of water content when the water condensate onto the dust particles. The mass ratio observed at outdoor ambient was used in the laboratory while incorporating the outdoor temperature and humidity. The condensation of water on the dust particles resulted in almost five times more water weight than the dust particles weight for the period of over six hours. Later, the solution excracted from the dust and water mixture was sampled for further analysis. The solution was poured onto the laser treated surface and it was left for drying. The dried solution on the workpiece surfaces was characterized using SEM and EDS. The force needed removing the dried solution from the workpiece surfaces was measured incorporating the scratch tests. To observe the influence of the dried liquid solution on the surface of the workpiece, the dried solution was cleaned completely by a desalinated water jet. Finally, the resulting surface was characterized.

## 3 Results and discussion

A laser surface processing of Ti6Al4V is considered while incorporating nitrogen as an assisted gas. The findings of the resulting surface characteristics and the dust particles influence on the laser treated surfaces are presented below.

### 3.1 Surface texture characteristics and wetting state

**Fig. 1:**
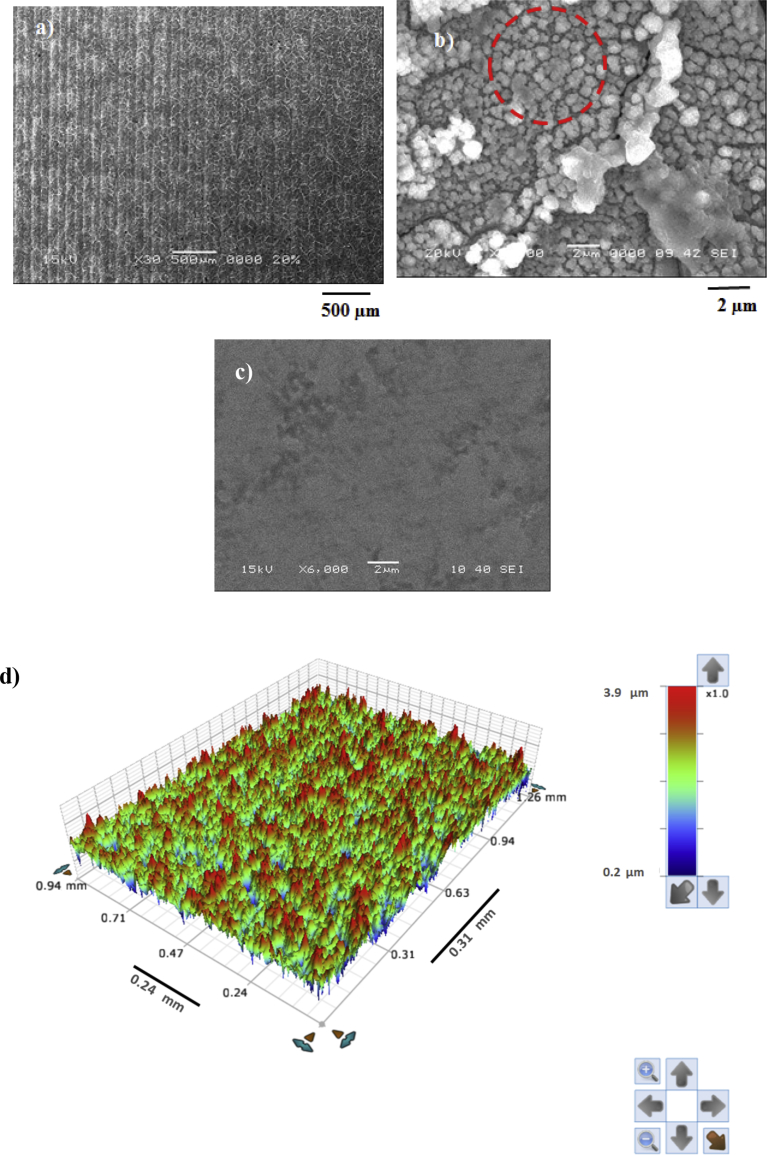
SEM micrographs of laser treated and as received, and 3D optical image of the laser treated surface: a) SEM micrograph showing regular scanning tracks, b) micro/nano size surface texture (dotted red circle), c) as received surface, and 6) 3D optical image of surface topology of laser treated surface.

Fig. 1 depicts SEM micro-image of the top surface of the workpiece. Regular and equally spaced laser scanning tracks are formed on the laser treated surface (Fig. 1(a)). The treated surface is free from the large size defects and excessive molten material flow among the tracks. The repetitive laser pulses at 1500 Hz frequency are responsible forming the regular scanning tracks; in which case, the irradiated spots overlap with 70% overlapping ratio. The intensity distribution of the beam is Gaussian on the surface; therefore, the beam power attains its peak at the center of the heated spot. This behavior results in evaporation of the surface at the center of the heated spot and the surface melts in the close section of spot edges. Consequently, the molten fluid partially fills the initially formed cavity during evaporation of the surface. Hence, the surface texture changes in such a way that micro/nano size pillars are formed (Fig. 1(b). The surface texture forms hierarchically distributed small size pillars covering the entire surface. This phenomenon can be observed from Fig. 1(c), in which 3D image is demonstrated.

**Fig. 2:**
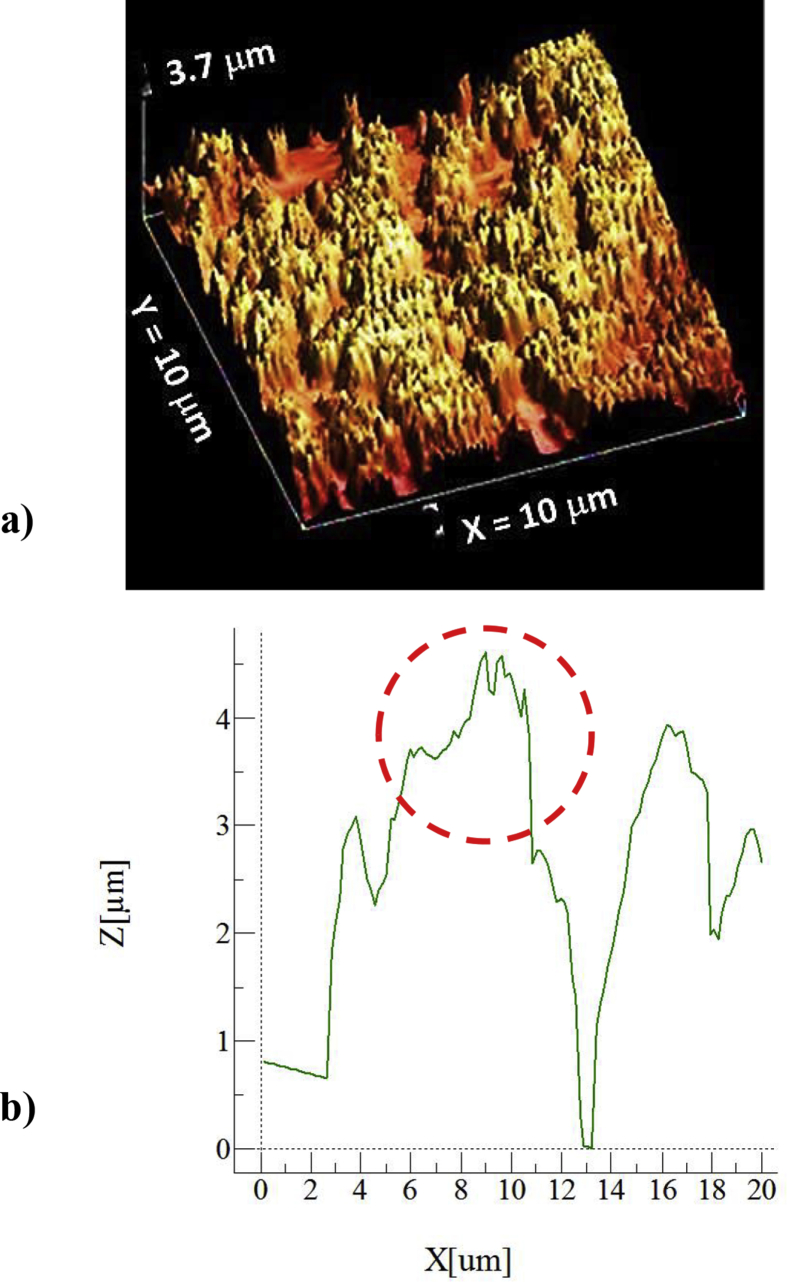
AFM data for laser processed surface: a) 3D-image of laser processed surface, and b) line scan along the surface (the red dotted circle demonstrates the presence of nano-size structures).

**Fig. 3:**
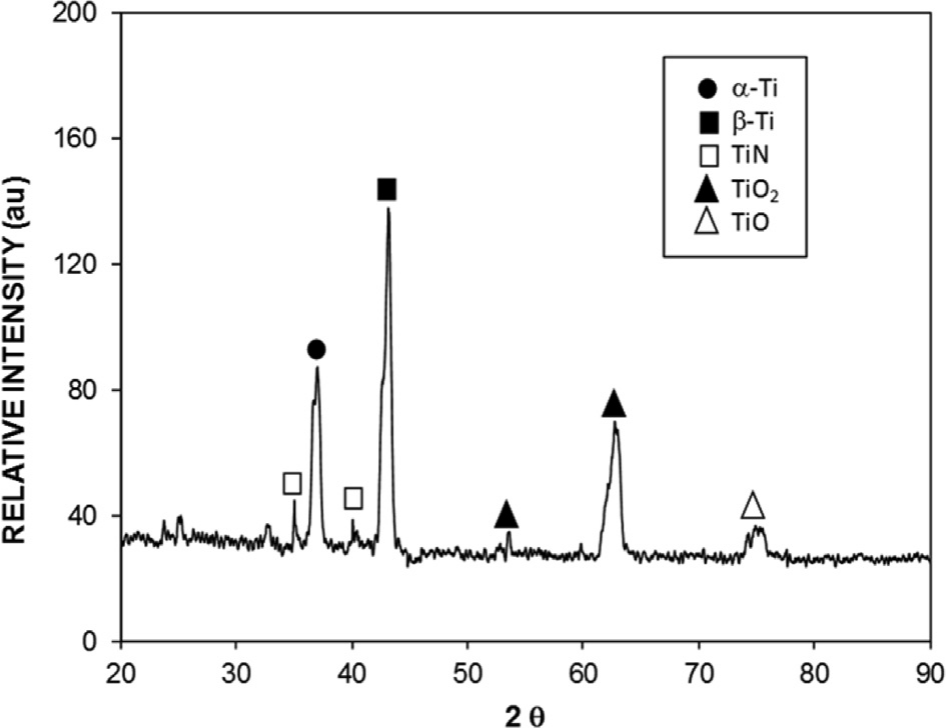
X-ray diffractogram of laser treated surface.

**Fig. 4:**
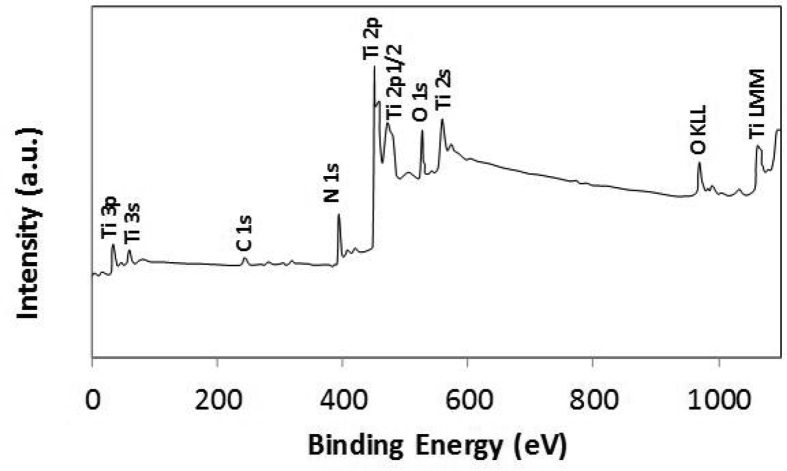
XPS spectra for the laser treated surface.

Fig. 2 shows atomic force microscopy (AFM) data for 3D image of the surface (Fig. 2(a)) and texture scan along the treated surface (Fig. 2(b)). The texture height is small within micrometer and nanometer sizes. The average roughness is about 3.2 μm on the surface while the roughness parameter is about 0.62. The roughness parameter corresponds to the ratio of the pillars area over the corresponding projected area on the surface. Moreover, inclusion of nitrogen during processing triggers the formation of the nitride species. This behavior is observed from Fig. 3, in which X-ray diffractogram of the workpiece surface is shown. TiN peak is evident (JCPDS 38-1420) and its formation can be related to: i) inward diffusion of nitrogen from surface, and ii) dissolution of nitrogen in the molten liquid; in which case, the high solidification rate in the molten surface gives rise to feathery like structure with TiN compounds. Nitrogen can diffuse into the vicinity of the surface and forms an interstitial nitrogen solid solution in α-titanium phase. As the nitrogen diffusion cross the limiting of nitrogen concentration in α-phase, a reaction is initiated while forming the nitride phase, i.e. the formation of phases can be formulated as α-Ti → α (N)-Ti → TiN. In addition, the peaks of TiO_2_ (JCPDS 21-1272) and TiO (JCPDS 85-2084) are observed in Fig. 3. In addition, XPS is realized for the laser treated surface and Fig. 4 shows XPS spectra for the laser treated surface. The binding energy of Ti 2p 455.5 eV demonstrates TiN bonding while the shifted peak of Ti 2p at 455.9 eV and O 1s at 531.5 eV demonstrates the Ti O bonding. Similarly, the Ti 2p1/2 peak at 464.7 eV corresponds to Ti O_2_. Moreover, the existence of oxygen in the assisting gas and/or initially surface oxidation before the laser processing is related to the formation oxide compounds at the treated surface. The nitride and oxide compounds can be assessed through Fourier-transform infrared spectroscopy (FTIR).

**Fig. 5:**
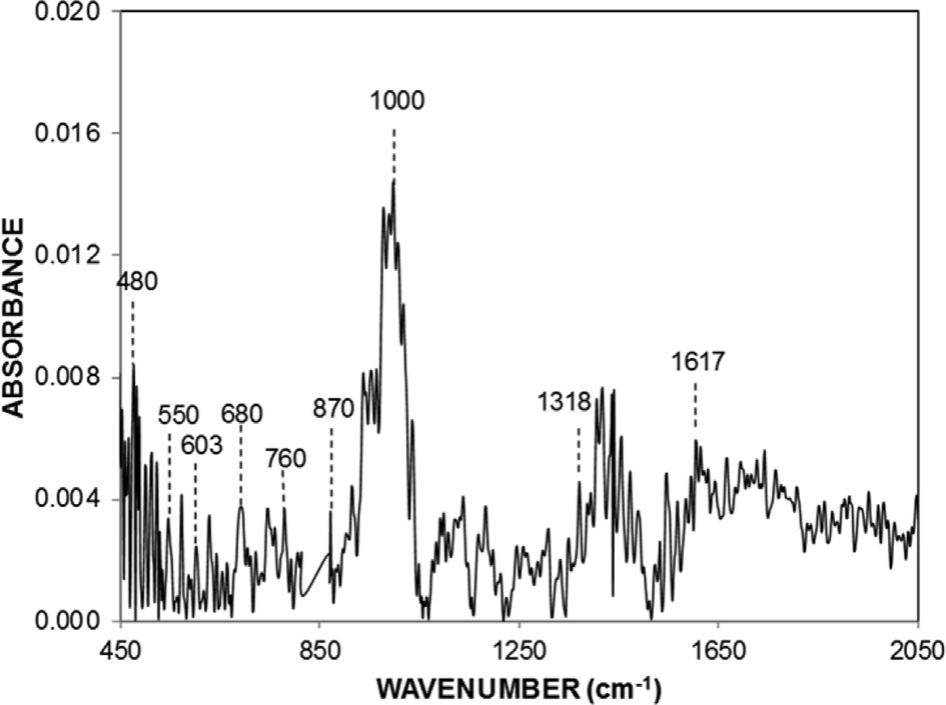
FTIR data of laser processed surface.

Fig. 5 depicts FTIR results for the processed surface. The peaks at 550 and 870 cm^−1^ corresponds to the Ti−O bonds [24]. The peak occurring at 680 cm^−1^ is attributed to the TiO_2_ in rutile structure [25]. The peak at 480 cm^−1^ is attributed to Ti−N vibrational level of the bond [25]. In addition, Ti−N bond stretches at 603 cm^−1^ [26]. The peak at 1618 cm^-1^ N−O−N represents antisymmetric stretching [26] and Ti−N−O appears at 1000 cm^−1^ [26]. The peak at 760 cm^−1^ is associated with vanadium. Table 2 gives the energy dispersive spectroscopy (EDS) data (wt%) for the treated and untreated surfaces. The surface is appeared to have almost uniform elemental distribution and the distribution does not vary notably from that of the as received surface. The presence of nitrogen is apparent in Table 2 even though the light elements are difficult to quantify because of the quantification errors associated. Nevertheless, the nitrogen concentration is because of TiN compound formed at the surface. The presence of oxygen is due to the oxide compounds formed at the surface. Because of the high temperature and high cooling rates involvement during the laser processing, the residual stress can be formed in the laser treated layer and the stress levels can be estimated from the XRD data. In this case, the calculations are conducted for TiN (111) planes ($2\theta =.36.6^{o}$) while incorporating the modules of elasticity of the laser nitrided sample, which is 450 GPa [21].

**Table 2:** EDS data (wt%) for the laser processed and untreated surfaces.

	Ti	Al	V	N	O
As Received	Balance	6.2	4.3	-	2.1
Laser Treated	Balance	5.9	4.1	8.2	9.1

**Fig. 6:**
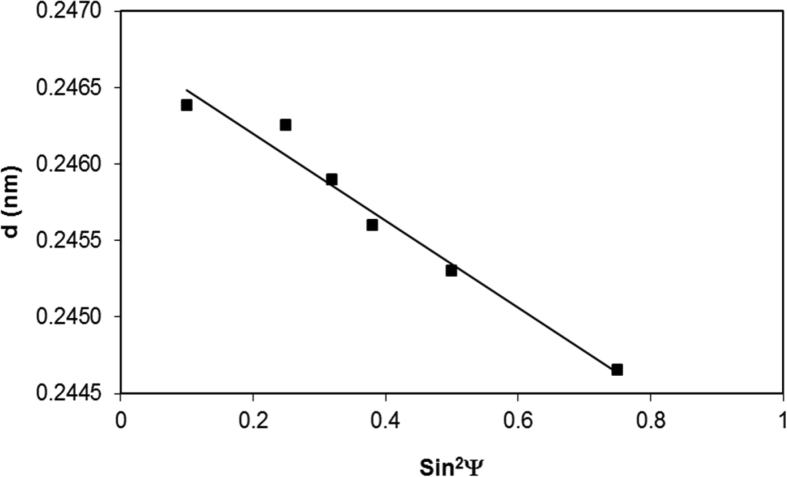
Dependence of d-spacing with tilt angle.

Fig. 6 shows the d-spacing with sin^2^Ψ, where Ψ is the tilt angle of the treated sample. The d-spacing varies with sin^2^Ψ in a linear form while indicating that the shear-strain developed in the processed layer is negligible [21]. Hence, the stress calculations (Eq. (1)) reveal that the residual stress developed is compressive and its value is about -410 ± 20 MPa. The error associated with the stress estimation is about ±20MPa. The microhardness at the laser treated surface (670 ± 20 HV) attains larger value than that of untreated surface (315 ± 10 HV). The microhardness enhancement of the surface is related to the high cooling rates during the solidification of the molten material on the surface. The nitride species in the laser treated surface also add to the microhardness increase.

The liquid droplet contact angle can be formulated using the Young's equation [27]:

(2) $$cos\theta =\frac{\left(\gamma_{sv}-\gamma_{sl}\right)}{\gamma_{lv}}$$

**Fig. 7:**
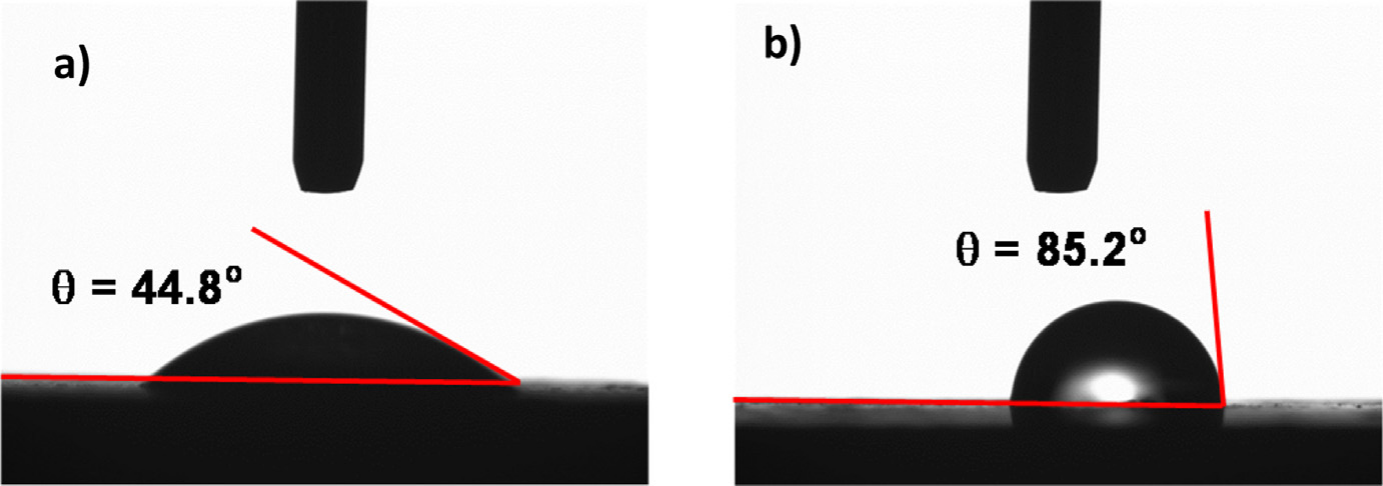
Water droplet contact angle on surfaces: a) untreated surface, and b) laser processed surface.

Where, *θ* represents the contact angle, γ_sv_ corresponds to the interfacial tensions of the solid-vapor phases, γ_sl_ represents the interfacial tensions of the solid-liquid, *γ*_*lv*_ corresponds to the interfacial tensions of the liquid-vapor phases. However, the Young's equation (Eq. (2)) is valid for the smooth surfaces and it can be corrected incorporating the roughness parameter (*r*). Hence, the modified equation due to the contact angle becomes [28]:

(3) $$cos\theta_{w}=\frac{r\left(\gamma_{sv}-\gamma_{sl}\right)}{\gamma_{lv}}$$

where *θ*_*w*_ corresponds to the contact angle of rough surface and *r* represents the roughness parameter. In Eq. (3), *r* = 1 represents smooth surface while *r* < 1 corresponds to rough surface. The further arrangements yield the equation for the contact as [29]:

(4) $$cos\theta_{c}=f_{1}cos\theta_{1}+f_{2}cos\theta_{2}$$

where *θ*_*c*_ is the apparent contact angle. In Eq. (4), the interfacial component of the liquid-solid phases is *f*_*1*_ and the interfacial component of the liquid-vapor phases is *f*_*2*_, the contact angle at the interface of the liquid-solid phases is *θ*_*1*_, and the contact angle at the interface of the liquid-vapor phases is *θ*_*2*_. However, in the case of the interface between air and liquid, *f*_*1*_ is expressed in the form of the fraction of solid (*f*_*1*_) and air fraction (*f*_*2*_), i.e. it yields (1 – *f*_*2*_). For the case *f*_*2*_ = 0 is the non-contacting droplet on the surface and *f*_*2*_ = 1, which corresponds to the surface being wetted. The surface wetting state can change from Cassie-Baxter state to Wenzel state based on the surface texture [30, 31]. The laser treated surface comprises of the combination of micro/nano pillars of different heights (Fig. 2), the laser treated surface demonstrates the hydrophilic wetting state. This behavior is attributed to the large pitch spacing between the pillars, which increases the size of the air pockets occupying the pillar gaps. Fig. 7 depicts the optical images of the droplets on the untreated and laser treated surfaces. The droplet contact angle is 44.8°±2° for as received workpiece and it is 85.2°±2° for the laser treated surface. Although the laser processing results in hydrophilic wetting state on the surface, the contact angle remains larger (θ = 85.2°±2°, where θ is the droplet contact angle) for the treated surface as compared to the as received surface (θ = 44.8°±2°).

The droplet contact angle takes almost same values on the laser treated surface and small differences in the contact angle is related to the variation of the surface texture characteristics such as pillars height and pillars gap across the surface. The droplet contact angle method is used to estimate the free energy of the laser processed surface [32]. The liquid droplets incorporated for the surface free energy assessment include water, glycerol, and diiodomethane. The arrangement of the formalism can be found in the early work [33, 34]. For the contact angle and surface energies, the following equation can be written [33, 34]:

(5) $${\text{γ}}_{L}\left(\cos\text{θ}+1\right)=2\sqrt{{\text{γ}}_{S}^{L}.{\text{γ}}_{L}^{L}}+2\sqrt{{\text{γ}}_{S}^{+}.{\text{γ}}_{L}^{-}}+2\sqrt{{\text{γ}}_{S}^{-}.{\text{γ}}_{L}^{+}}$$

here, γ_L_ corresponds to the surface tension of the liquid phase, γ_S_ is the solid surface free energy, γ_SL_ is the interfacial solid-liquid free energy, θ is the droplet contact angle. In addition, γ^+^ and γ^−^ correspond to the electron acceptor and donor parameters related to the free energy due to the solid and liquid phases while *S* and *L* represent the solid and liquid. Eq. (5) is used determining the free surface energy of laser treated workpieces, i.e. the values of $\gamma_{S}^{L}$, $\gamma_{S}^{+}$, and $\gamma_{S}^{-}$ can be determined. The data for $\gamma_{L}^{L}$, $\gamma_{L}^{+}$, and $\gamma_{L}^{-}$ are presented in Table 3 [23]. In this case, several repeats are conducted for contact angle measurements assuring the repeatability of the measurements in accordance with the early study [23]. Adopting Eq. (5), the surface energy for the laser processed surface is 48.9 mJ/m^2^ and it is similar order of the TiN coated surface (51 mJ/m^2^) [35]. However, surface free energy of the as received titanium alloy is also obtained from the contact angle measurement technique. It is found that the surface free energy is in the order of 56 mJ/m^2^, which is close to that reported in the early work (56 mJ/m^2^ [36]).

**Table 3:** Data used in surface energy measurements [30].

	γ_L_ (mJ/m^2^)	$\gamma_{L}^{L}$ (mJ/m^2^)	$\gamma_{L}^{+}$ (mJ/m^2^)	$\gamma_{L}^{-}$ (mJ/m^2^)
Water	72.8	21.8	25.5	25.5
Glycerol	64	34	3.92	57.4
Diiodomethane	50.8	50.8	0.72	0

### 3.2 Dust effects on treated surface

**Fig. 8:**
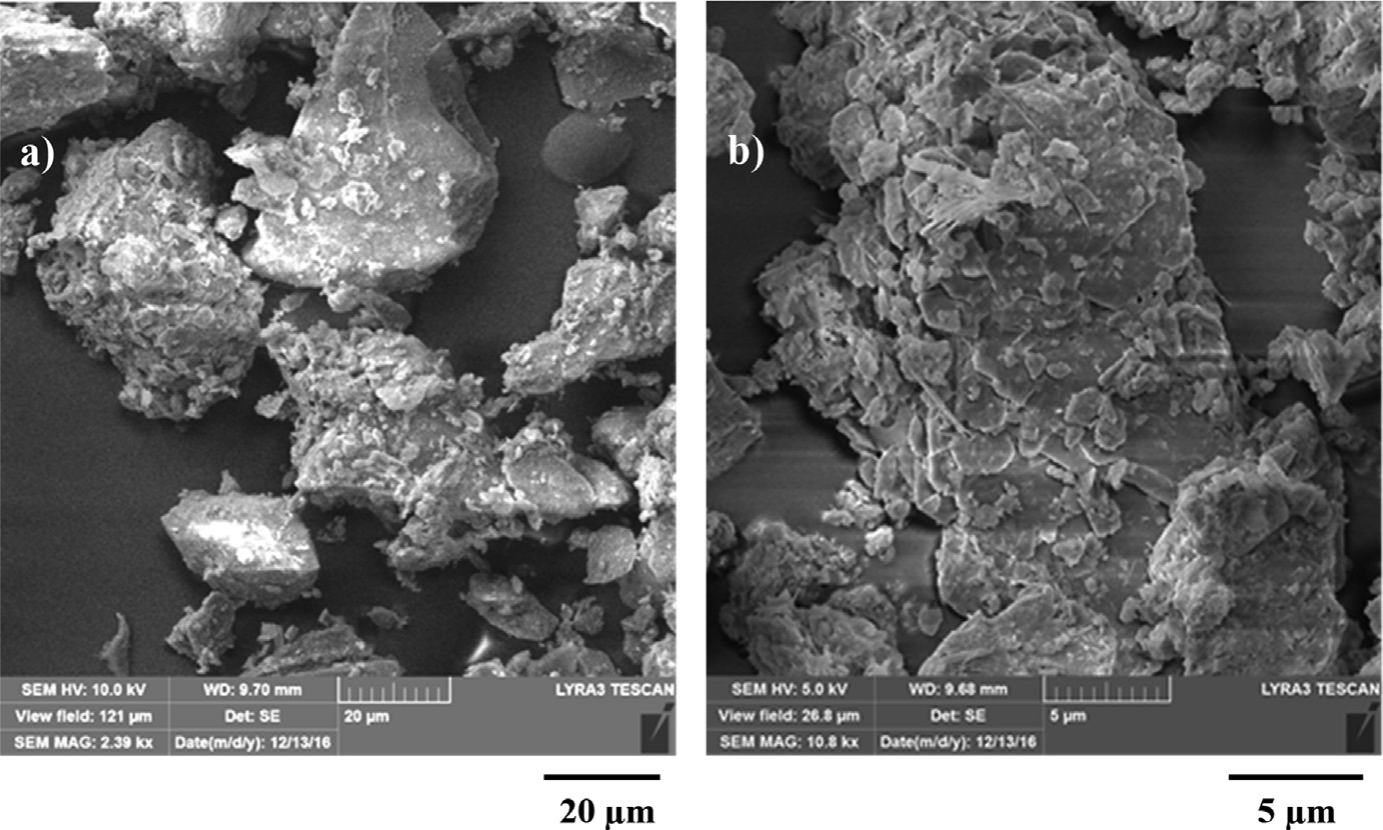
SEM micrographs of dust particles: different shapes and sizes, b) small size dust attaching at dust particles surface.

Fig. 8 depicts SEM micro-images of the dust particles collected. The particles compose of different geometries with the average size of 1.2 μm. The particles with small sizes attach at the large size particle surfaces while forming the clusters (Fig. 7(b)). This is related to the long duration of suspension of the small size particles in the air while interacting with the solar rays [7].

**Fig. 9:**
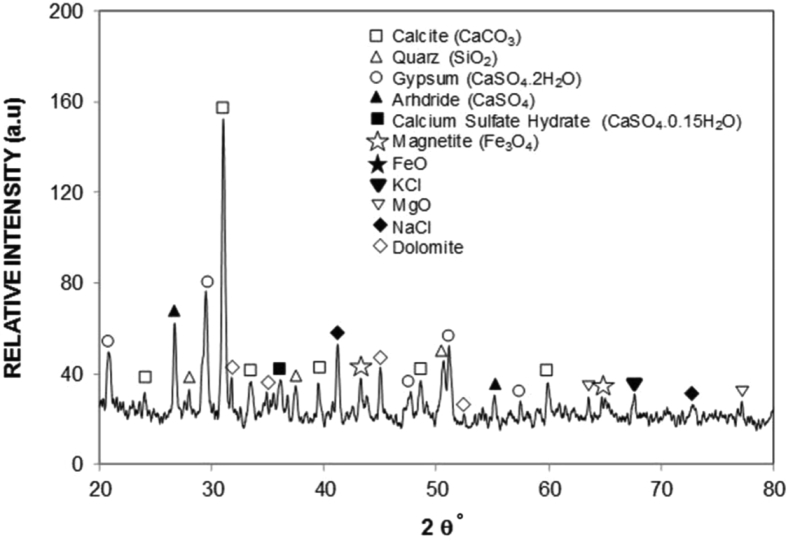
X-ray diffractogram of dust.

Fig. 9 depicts the particles X-ray diffractogram while Table 4 gives the elemental composition (wt%) of the dust particles. The particles consist of different elements such as Ca, K, Na, S, Si, O, Mg, Fe, and Cl. The inclusion of the different elements in the particles is related to the geologic-structures of the desert environment. The iron peaks in X-ray diffractogram overlap with Al and Si peaks and S peak represents the anhydrite or gypsum (CaSO_4_) components, and Fe appears in relation to the clay-aggregated hematite (Fe_2_O_3_). In humid environments, water condensates onto the dust particles. Some dust compounds dissolve into the condensed water. However, to mimic the outdoor condensation of water in humid air conditions, the tests are conducted in the humid days. It is found that the condensate of water onto the dust particles has the weight ratio of almost 1/5 (1 represents the dust particles while 5 corresponds to water condensate). Based on the outdoor condensation tests, distilled water is mixed with the dust particles for one hour and the liquid solution, which is the mixture of distilled water with the dissolving components of the particles, is extracted for further examinations. The alkalinity (pH) of the extracted solution rises to 8.6 indicating high basic content, which is attributed to the mixing of dissolved compounds in distilled water. The solution is poured onto the laser treated surface, which is placed in a drying environment for six hours in the outdoor condition.

**Table 4:** Composition (wt%) of dust and dried liquid solution.

	Si	Ca	Na	S	Mg	K	Fe	O
Dust	12.2	8.1	2.4	2.2	1.8	1.2	1.1	Balance
Dried Liquid Solution	0.2	7.5	2.1	-	-	0.9	0.4	Balance

**Fig. 10:**
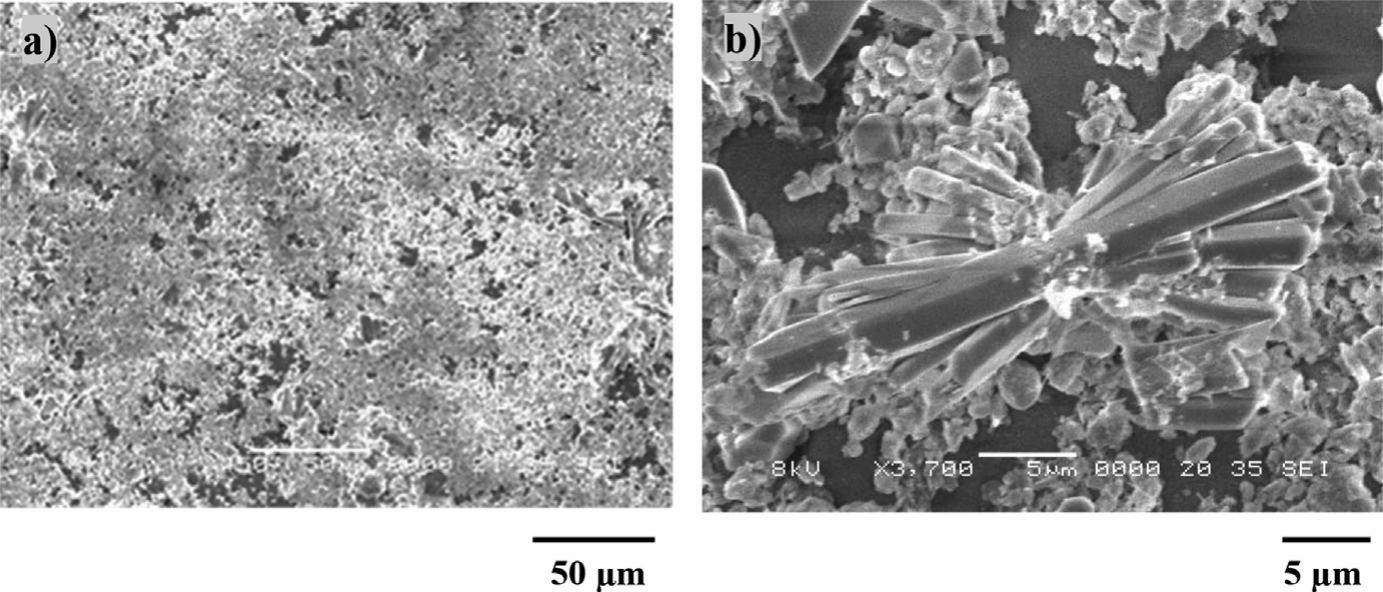
SEM micrographs of dried solution: a) dried liquid solution covering laser processed surface, and b) crystallized dried solution on laser processed surface.

Fig. 10 depicts SEM image of dried solution on the laser treated surface. The EDS analysis is conducted determining the elemental composition of the dried solution. Table 4 gives the EDS data (wt%) of the dried liquid solution. The SEM micrograph reveals (Fig. 9) that various sizes of crystals are formed. In addition, the dried liquid solution comprises of different elements, i.e. Na, Ca, K, Cl, O, and Si (Table 4). However, the molar ratio of Cl and Na, or Cl and K, or Ca and Cl do not evolve in a stoichiometric ratio. The presence of crystals on the surface is associated with non-stoichiometric compounds in the dust particles. To determine the wetting state of the solution on the workpiece surface, the solution is poured onto both the textured and untreated surfaces and the spreading coefficient for the solution is obtained. The interfacial tension plays a critical role for solution spreading onto the workpiece surfaces. In association with the early study [37], the spreading coefficient can be written in the form of $S_{Liquid\left(a\right)}=\gamma_{la}-\gamma_{sa}-\gamma_{sl}$, where γ_la_ represents the surface tension of the solution in air, and γ_sa_ corresponds to the surface energy of the workpiece in air, and $\gamma_{sl}$ represents the interfacial tension across the solution and the workpiece. Once the spreading coefficient becomes less than zero, the solution spreads and wets the entire workpiece surface. The surface tension of the solution is determined from the capillary method [38] and the surface tension is estimated about 74 mJ/m^2^, i.e. it is similar order of the water surface tension (0.072 N/m).

**Fig. 11:**
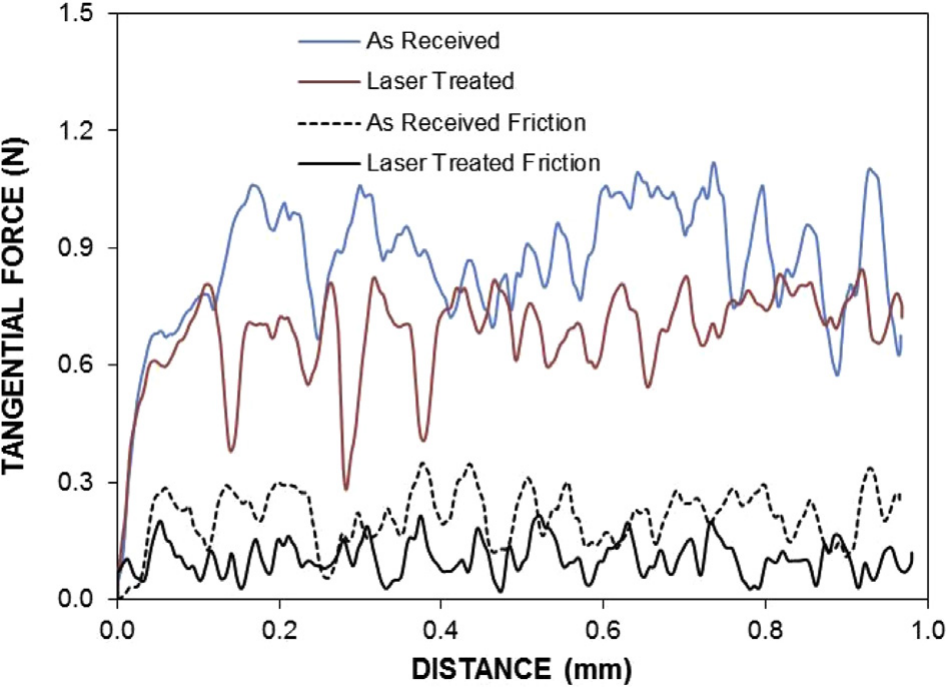
Tangential and frictional forces along the scratch distance. Tangential force represents force needed removing dried solution from surface and frictional force is due to laser processed and untreated surfaces without dried solution presence.

**Fig. 12:**
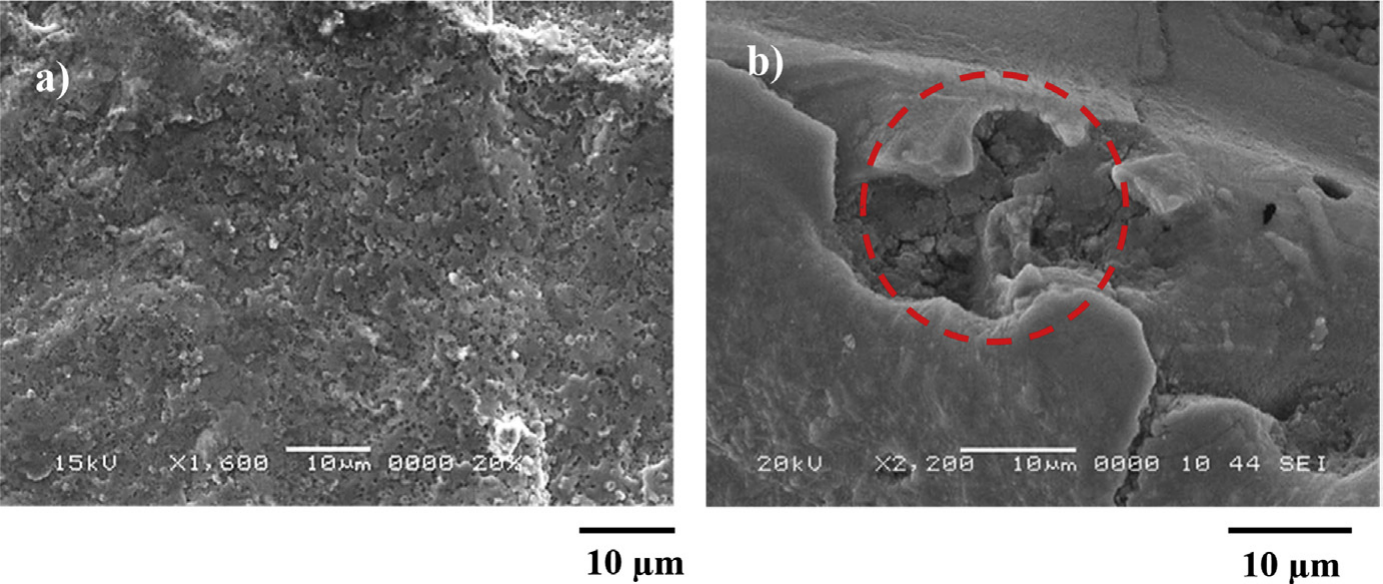
SEM micrographs of surfaces after removal of dried solution from surface by desalinated water jet: a) laser processed surface, and b) as received surface and shallow pit site is evident (marked in a dotted circle).

Hence, the interfacial tension across the workpiece surface (treated and untreated surfaces) and the solution is considered to be same as that of the solid surface and water. Using the Young's equation (Eq. (3)), the interfacial tension across the workpiece surface and the water can be obtained. This arrangement yields $\gamma_{sl}=\gamma_{sa}-\frac{1}{r}\;\gamma_{la}cos\theta_{w}$, i.e. the interfacial tension (*γ*_*sl*_) becomes 38.15 mJ/m^2^ (here, *γ*_*sa*_ = 48.9 mJ/m^2^, *γ*_*la*_ = 74 mJ/m^2^ *r* = 0.62, and *θ*_*w*_ = 85.2°). It should be noted that *r* is the roughness parameter. The spreading coefficient for the solution ($S_{Liquid\left(a\right)}=\gamma_{la}-\gamma_{sa}-\gamma_{sl}$), on the laser processed workpiece surface, yields – 13.05 mJ/m^2^. The spreading coefficient remains negative, i.e. $S_{Liquid\left(a\right)}<0$. In addition, the adhesion force removing the dried mud from the laser treated and untreated surfaces can be obtained via incorporating the micro-scratch tests. Fig. 11 shows tangential and frictional forces obtained from the micro-scratch tester. The tangential force represents the force needed towards removing the dried liquid from the workpiece surface (treated and untreated). The frictional force for the solid surface is also included in the Fig. 11. The tangential force becomes larger than the frictional force on the laser treated surface; however, this behavior is true almost five times larger than the frictional force for untreated surface. Hence, the dried solution bonds strongly on both textured and untreated surfaces. The adhesion force is considerably large for untreated surface. The laser treatment reduces the bonding of the dried solution on the workpiece surface. Consequently the efforts needed for eliminating the dried solution becomes less for the laser processed surface. Alternatively, the efforts required cleaning the surface from the dust particles in the humid air conditions, through the mechanical method (such as rubbing the surface), remain extremely larger as compared to the removal of the dust particles in the dry air conditions. Moreover, the dried solution is cleaned from the surfaces by a pure water jet towards assessing the effects of the dried solution on the surfaces. The water jet cleaning is continued to ensure all the residues is removed. Fig. 12 shows SEM micro images of processed (Fig. 12(a)) and untreated (Fig. 12(b)) surfaces after dried solution being removed. Local pit sites are noted on untreated surface and the pits remain shallow. In the case of the laser treated surface, pitting is not observed. This is related to the formation of passive layer on the surface through development of the dense layer and nitride species in the surface region after the laser textured process, which is consistent with the previous study [3].

## 4 Conclusion

Laser processing of Ti6Al4V alloy surface is carried out and texture and wetting characteristics of the resulting surface are analyzed. The wetting state and the energy of the treated surface are assessed incorporating the contact angle measurement technique. The characteristics of the dust particles in terms of size, shape, and composition are presented. The solution formed onto the dust particles, dust due to water condensation in humid air ambient, is examined and the dried liquid solution is characterized. The adhesion of the dried solution on untreated and the laser textured surfaces is evaluated via measuring the force needed removing the dried solution from the workpiece surfaces. The effects of the dried solution on untreated and the treated surfaces are also considered. The conclusions derived from the present work are listed below:

- Hierarchically distributed micro/nano pillars, which is because of the surface combination of melting and evaporation across the heated spot. The laser treatment gives rise to asperity free surface in terms of micro-cracks and large size voids. Because of the large gap size in between the pillars, the laser treated surface remains hydrophilic; however, the water droplet contact angle for the laser processed surface is larger than of the untreated surface.
- The nitride species are formed on the laser surface. The surface free energy of the laser treated workpieces is estimated as 48.9 mJ/m^2^, i.e. it is almost same as that of TiN. The environmental dust particles have various sizes and shapes. The average dust particle size is about 1.2 μm.
- The dust composes of different elements and some of which can dissolve in water condensate in the humid air ambient. This in turn develops a chemically active solution spreading onto the solid surface. The solution forms a liquid film on the laser treated and as received surfaces because of the spreading coefficient ($S_{Liquid\left(a\right)}<0$).
- The tangential force needed for removing the dried solution from the laser treated surface is significantly large because of the strong bonding between the dried solution and the workpiece surface. However, this is more pronounced for the untreated surface. Consequently, the laser processing of the alloy surface provides easiness for the mechanical cleaning of the settled dust particles from the surfaces in the humidity air ambient.
- Since the solution is chemically active, the pits sites are formed on as received surfaces; however, pitting does not occur for the laser treated surface. This is related to the passive layer formed on the laser treated surface.

The present study gives insight into the environmental dust effects on the laser treated Ti6Al4V alloy and findings are very useful towards assessing the laser textured surfaces and the environmental dust.

## Declarations

### Author contribution statement

Bekir S. Yilbas, Haider Ali, Abdullah Al-Sharafi, Hussain Al-Qahtani: Conceived and designed the experiments; Performed the experiments; Analyzed and interpreted the data; Wrote the paper.

### Funding statement

This work was supported by King Fahd University of Petroleum and Minerals (IN171001).

### Competing interest statement

The authors declare no conflict of interest.

### Additional information

No additional information is available for this paper.
